# Anti-tumor effects of a human VEGFR-2-based DNA vaccine in mouse models

**DOI:** 10.1186/1479-0556-7-10

**Published:** 2009-06-21

**Authors:** Ke Xie, Rui-Zhen Bai, Yang Wu, Quan Liu, Kang Liu, Yu-Quan Wei

**Affiliations:** 1 State Key Laboratory of Biotherapy and Cancer Center, West China Hospital, West China Medical School, Sichuan University, Guo Xue Xiang, No 37, Chengdu, Sichuan 610041, PR China

## Abstract

**Background:**

Vascular endothelial growth factor (VEGF) and its receptor, VEGFR-2 (Flk-1/KDR), play a key role in tumor angiogenesis. Blocking the VEGF-VEGFR-2 pathway may inhibit tumor growth. Here, we used human VEGFR-2 as a model antigen to explore the feasibility of immunotherapy with a plasmid DNA vaccine based on a xenogeneic homologue of this receptor.

**Methods:**

The protective effects and therapeutic anti-tumor immunity mediated by the DNA vaccine were investigated in mouse models. Anti-angiogenesis effects were detected by immunohistochemical staining and the alginate-encapsulate tumor cell assay. The mechanism of action of the DNA vaccine was primarily explored by detection of auto-antibodies and CTL activity.

**Results:**

The DNA  vaccine  elicited  a  strong, protective and therapeutic  anti-tumor  immunity  through  an anti-angiogenesis mechanism  in mouse models, mediated  by  the  stimulation of an antigen-specific response against mFlk-1.

**Conclusion:**

Our study shows that a DNA vaccine based on a xenogeneic homologue plasmid DNA induced autoimmunity against VEGFR-2, resulting in inhibition of tumor growth. Such vaccines may be clinically relevant for cancer immunotherapy.

## Background

Angiogenesis plays an important role in the growth, invasion and metastasis of most solid tumors [1-3]. Vascular endothelial growth factor (VEGF) and its receptor, VEGFR-2 (Flk-1/KDR), play a key role in tumor angiogenesis[4]. VEGFR-2 has a strong tyrosine kinase activity and mediates the transduction of major signals for angiogenesis[5]. Blocking the VEGF-VEGFR-2 pathway may inhibit tumor growth.

It is possible that breaking the immune tolerance to VEGFR-2 (Flk-1) on autologous angiogenic endothelial cells may enable tumor therapy through active immunity. However, immunity to angiogenic vessels is difficult to elicit with a vaccine based on autologous molecules because of the immune tolerance acquired during the early development of the immune system. Many genes have been highly conserved during the evolutionary process, which is evident from the degree of gene similarity among different species[6]. Sequence comparison using the SwissProt database indicates that the primary sequence of murine VEGFR-2 (Flk-1) is 85% identical to the human receptor (KDR) sequence at the amino acid level. Here, we investigate the feasibility of cancer immunotherapy using a vaccine based on a xenogeneic homologue of VEGFR-2 as a model antigen to break the immune tolerance against VEGFR-2 through a cross-reaction between the xenogeneic homologue and the self-molecule.

Anti-cancer vaccines have been extensively studied in animal models and clinical trials. Anti-cancer vaccine strategies have included immunization with whole-tumor cell vaccines, peptide vaccines, dendritic cell (DC) vaccines, viral vector vaccines, vaccines combined with adoptive T-cell therapy, and plasmid DNA vaccines[7]. Plasmid DNA vaccines are attractive because they are relatively easy to engineer and produce, and are safe to administer to humans [8-10]. A number of studies have reported the use of plasmid DNA-based vaccines for eliciting anti-tumor immunity in mice [11-14].

The current generation of plasmid DNA-based cancer vaccines may fail to elicit effective anti-tumor immunity because they do not trigger sufficient systemic activation of innate immunity. Complexing cationic liposomes to the plasmid DNA offers a relatively simple means of accomplishing this, as some researchers have shown that liposome-DNA complexes (LDCs) are extremely potent activators of innate immunity [14-16].

Here, we immunized tumor-bearing mice with human VEGFR-2 LDC vaccines to test their ability to induce anti-tumor immunity. We demonstrated that human VEGFR-2 LDC immunization elicited enhanced anticancer activity in these mice. The human DNA vaccine mediated its effect by impairing the formation of new tumor blood vessels through an anti-angiogenesis mechanism. The human VEGFR-2 DNA vaccine was well tolerated by all mice.

## Methods

### Plasmid construction and preparation of plasmid DNA

We constructed the expression vectors, pORF-Flk-1, by inserting the cDNAs encoding extracellular human or murine Flk-1 into the pORF-mcs between the NcoI (5') and NheI (3') restriction sites. These constructs were verified by nucleotide sequencing, and by protein expression on western blots following transient transfection into COS-7 cells. Large-scale plasmid DNA preparation was performed using an EndoFree™ Plasmid Giga kit (Qiagen, Chatsworth, CA).

### Animals and cell lines

BALB/c mice were purchased from the West China Experimental Animal Center. Murine breast carcinoma cell line 4T1, colon carcinoma cell line CT26 and endothelial cell line MS1 were purchased from the American Type Culture Collection. The MS1, COS-7 and breast carcinoma 4T1 cell lines were cultured in DMEM, and the colon carcinoma CT26 cell line was cultured in RPMI 1640, each supplemented with 10% (v/v) fetal bovine serum.

Animal protocols for these experiments were approved by the West China Hospital Cancer Center's Animal Care and Use Committee at the National Jewish Medical and Research Center.

### Preparation of liposome-DNA complexes

The cationic lipid DOTAP was mixed with the neutral lipid cholesterol at equimolar concentrations. The mixed powdered lipids were dissolved in AP-grade chloroform in a 100-ml round-bottomed flask, rotated on a Buchi rotary evaporator at 30°C for 30 min to make a thin film, then dried under vacuum for 15 min. The film was hydrated in 5% dextrose in water (D5W) to give a final concentration of 13 mM DOTAP and 13 mM cholesterol, referred to as 13 mM DOTAP:chol. The hydrated lipid film was rotated in a water bath at 50°C for 45 min and then 35°C for 10 min. The mixture was allowed to stand in the parafilm-covered flask at room temperature overnight, after which the mixture was sonicated at low frequency (Lab-line, TransSonic 820/H) for 5 min at 50°C, transferred to a tube, and heated for 10 min at 50°C. The mixture was sequentially extruded through Millipore (Billerica, MA) polycarbonate membranes of decreasing size (0.2 μm for 5 times and then 0.1 μm for 3 times) using a syringe. Liposome-DNA complexes (LDCs) were formed just before injection by gently mixing cationic liposomes with plasmid DNA at a ratio of 1:3 (w/w) in 5% aqueous dextrose at room temperature. The DNA:liposome mixture thus prepared was precipitate free and used for all the in vivo experiments. The size of the DNA fragments in the DNA:liposome mixture was determined to be in the range of 300–325 nm. (The preparation of liposome DNA complexes was supported by The Chemical Laboratories of the State Key Laboratory for Biotherapy).

### Therapeutic anti-tumor immunity and tumor models

Experiments with CT26 and 4T1 tumors were carried out in female BALB/c mice, 6–8 weeks of age. Tumors were established by sub-cutaneous (s.c.) injection of 2 × 10^5 ^CT26 tumor cells or 8 × 10^5 ^4T1 tumor cells in the right flank. Treatments were initiated 13 days post-tumor inoculation (CT26 colon carcinoma model) or 4 days post-tumor inoculation (4T1 breast tumor model), at a time when tumor nodules were palpable. Tumor dimensions were measured every 3 days using calipers, and volumes were calculated according to the following formula: width^2 ^× length × 0.52. The lung metastatic nodules were counted using a dissecting microscope[17]. Mice were immunized with 20 μg per injection per mouse of pORF-hFlk-1 LDC, pORF-mFlk-1 LDC, pORF-mcs LDC alone or with 5% dextrose (GS)[18]. Intravenous (i.v.) injections were performed once a week for 6 weeks. Due to the death of mice, the tumor volumes on day 28 (CT26 tumors) and day 34 (4T1 tumors) in the control groups only came from the surviving mice.

### Protective anti-tumor immunity and tumor models

At 6–8 weeks of age, female BALB/c mice were immunized with 20 μg per injection per mouse of pORF-hFlk-1 LDC, pORF-mFlk-1 LDC, pORF-mcs LDC alone or GS. Injections were performed by i.v. routes once a week for 6 weeks. Seven days after the last immunization, the mice were challenged with 2 × 10^5 ^CT26 tumor cells or 8 × 10^5 ^4T1 tumor cells by s.c. injection in the right flank.

### ELISA

96-well plates were coated with cell lysates (100 μL/well) in coating buffer (carbonate-bicarbonate, pH 9.6) overnight at 4°C. Plates were washed with PBST (0.05% Tween 20 in PBS) and blocked for 1 hour at 37°C with 100 μL/well of 1% bovine serum albumin (BSA) in PBST. Mice sera collected at 0, 2nd, 4th, 6th weeks immunization, diluted serially in PBS were added for 2 hours at 37°C, washed and followed by a dilution of anti-mouse secondary antibody conjugated to horseradish peroxidase. Enzyme activity was measured with an enzyme-linked immunosorbent assay (ELISA) reader (Bio-Rad Laboratories, Hercules, CA).

### Western blot analysis

Western blot analysis was performed as described[19,20]. Briefly, lysates of cells were separated by SDS-PAGE and gels were transferred onto PVDF membranes by electroblotting. The membranes were blocked in 5% (w/v) nonfat dry milk, washed, and probed with mouse sera at 1:200. Blots were then washed and incubated with an HRP-conjugated secondary antibody (1:6000–10,000), and visualized with chemiluminescence reagents.

### Immunohistochemistry

To explore whether the anti-tumor immunity involved the inhibition of angiogenesis, vessel density in the tumor tissue, and angiogenesis in vivo, were determined as described previously[18]. Frozen sections were used to determine vessel density with an anti-CD31 antibody.

### Alginate-encapsulate tumor cell assay

Mice were immunized as above. Alginate beads containing 1 × 10^5 ^tumor cells (CT26 tumor cells) per bead were implanted s.c. into both dorsal sides of the immunized mice. After 12 days, the mice were injected i.v. with 0.1 ml of a 100 mg/kg FITC-dextran (Sigma) solution. Twenty minutes after FITC-dextran injection, alginate beads were photographed after being exposed surgically and then rapidly removed. The uptake of FITC-dextran was measured as described[18,21].

### CTL assays

The possibility that hFlk-1-specific cytotoxicity was mediated by cytotoxic T lymphocytes (CTLs) was determined by a ^51^Cr release assay as described previously[22,23]. Briefly, BALB/c mice were immunized with 20 μg DNA as described above. Spleens were collected on day 7 after the last vaccination. T lymphocytes were isolated from single-cell suspensions with using a Nylon Fiber Column T (L-Type, WAKO) to use as CTL effector cells; MS1 murine endothelial cells, which express mFlk-1[24], were used as target cells. Effector and target cells were seeded into a 96-well microtiter plate at various effector/target ratios. The CTL activity was calculated by the following formula: %lysis = [(experimental release - spontaneous release)/(maximum release - spontaneous release)] × 100.

### Evaluation of possible adverse effects

Potential toxic effects of the vaccines in immunized mice were investigated for more than 5 months. Gross measures such as weight loss, ruffling of fur, life span, behavior, and feeding were investigated. Tissues from the major organs (heart, liver, spleen, lung, kidney, ovary and so on) were fixed in a 10% neutral buffered formalin solution, embedded in paraffin and 3 to 5 μm sections were stained with hematoxylin and eosin (H&E). Animals were subjected to complete peripheral blood counts and differentials, and biochemical tests.

### Statistical analysis

Survival curves were generated by the log-rank test. The statistical significance of results in all of the experiments was determined by Student's t test and ANOVA. *P *value < 0.05 was considered statistically significant.

## Results

### Induction of therapeutic and protective anti-tumor immunity

**Figure 1:**
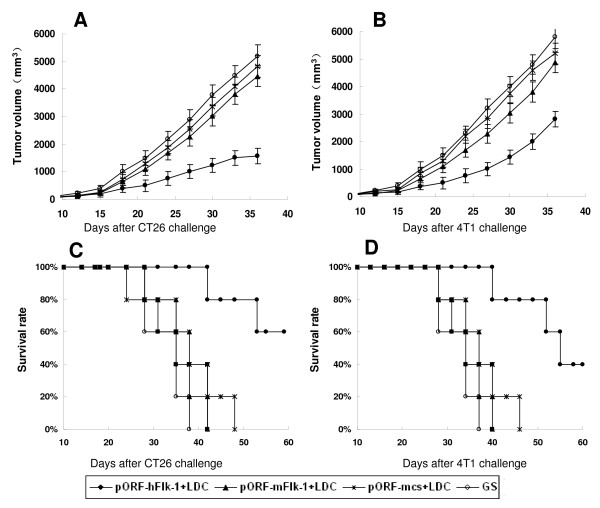
**Induction of therapeutic anti-tumor immunity**. Mice (five per group in both the CT26 and 4T1 tumor models) were treated i.v. once a week for 6 weeks with 20 μg of pORF-hFlk-1 LDC, pORF-mFlk-1 LDC, pORF-mcs LDC alone or GS. Treatment was started 13 days after 2 × 10^5 ^CT26 cells were subcutaneously administered to the mice, (A, C) or 4 days after they received 8 × 10^5 ^4T1 tumor cells (B, D). Data are expressed as means ± SEM. A significant increase in survival in human VEGFR-2 LDC-treated mice, compared with the control groups (*P *< 0.01, by log-rank test), was found in both tumor models.

To explore the therapeutic efficacy of human VEGFR-2 LDC, we treated mice either 13 days (CT26 tumors) or 4 days (4T1 tumors) after the implantation of tumor cells, when the tumors were palpable. Treatment with human VEGFR-2 LDC once weekly resulted in significant anti-tumor activity in both the CT26 colon carcinoma (CT26) and 4T1 breast tumor (4T1) models. Survival of the tumor-bearing mice treated with human VEGFR-2 LDC was also greater than that of the controls (Figure 1).

**Figure 2:**
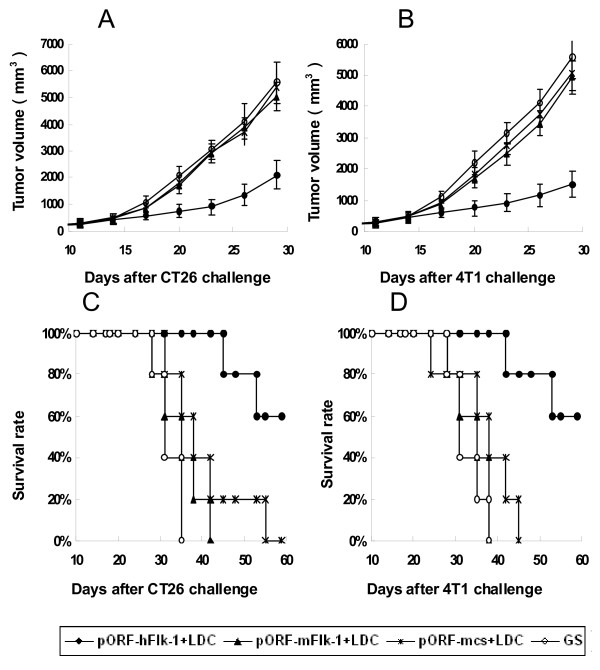
**Induction of protective anti-tumor immunity**. Mice (five per group) were immunized with 20 μg of pORF-hFlk-1 LDC, pORF-mFlk-1 LDC, pORF-mcs LDC or GS once a week for 6 weeks. Mice were then challenged subcutaneously with 2 × 10^5 ^CT26(A, C) tumor cells, or 8 × 10^5 ^4T1 tumor cells(B, D), 1 week after the sixth immunization. There was an apparent difference in tumor volume between human VEGFR-2 LDC-immunized and control groups. Results are expressed as means ± SEM.

Mice were immunized i.v. once a week for 6 weeks with human VEGFR-2 LDC (pORF-hFlk-1 LDC), and then were challenged with 2 × 10^5 ^CT26 tumor cells or 8 × 10^5 ^4T1 tumor cells. Controls included mice that were not vaccinated (GS), and mice immunized i.v. with LDC containing either non-coding plasmid DNA or mouse VEGFR-2 DNA (pORF-mcs LDC alone or pORF-mFlk-1 LDC). Tumors grew progressively in all non-immunized mice and in mice immunized with pORF-mFlk-1 LDC or pORF-mcs LDC alone, but there was an apparent protection from tumor growth in mice immunized with pORF-hFlk-1 LDC (Figure 2).

**Figure 3:**
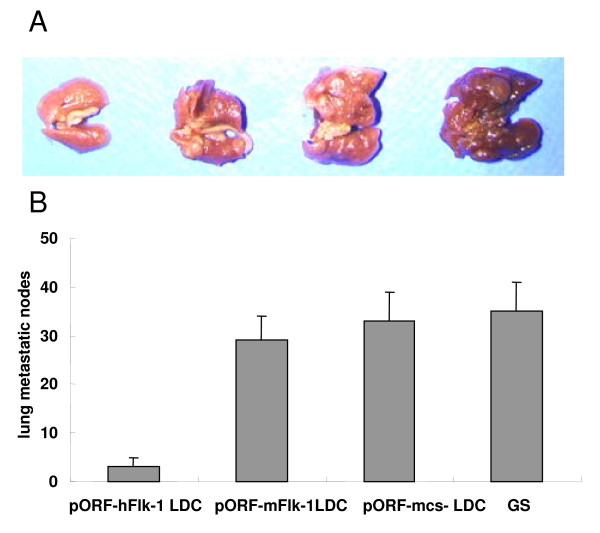
**Protection against pulmonary metastases**. BALB/c mice (4T1 tumor model) were treated i.v. once a week for 6 weeks with 20 μg LDC beginning 4 days after subcutaneous challenge with 8 × 10^5 ^4T1 tumor cells. Top(A): representative lung metastatic nodule specimens from mice challenged with 4T1 breast tumor cells; bottom(B): average numbers of lung metastatic nodules. The lungs from pORF-hFlk-1-treated mice showed significant differences compared with other groups (P < 0.05 or P < 0.01). Columns: mean lung metastatic nodules (n = 5); bars: SD.

We observed a marked reduction in the dissemination of pulmonary metastases in all experimental animals following six immunizations with human VEGFR-2 LDC in the 4T1 breast tumor model (Figure 3).

**Figure 4:**
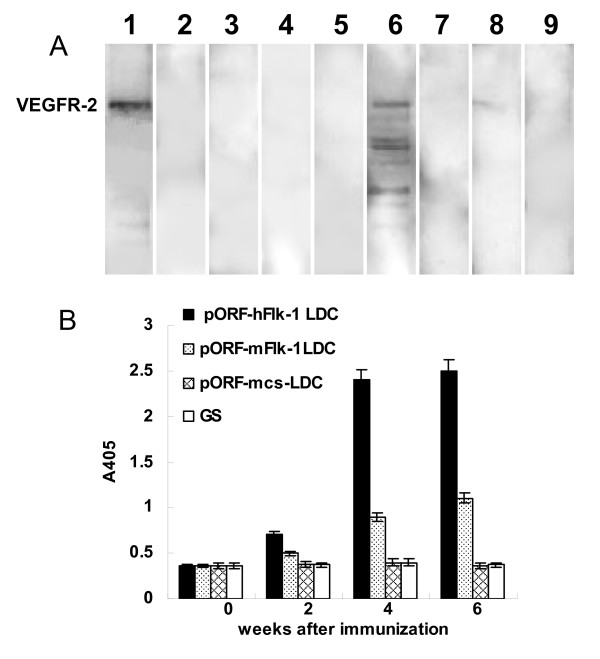
**Characterization of the auto-antibodies**. Western blotting(A) and ELISA(B) showed that the VEGFR-2 protein was recognized by the sera isolated from mice immunized with human VEGFR-2 LDC, but not control sera. Lane 1 = positive control. Lanes 2–5 = samples obtained prior to immunization. Lanes 6–9 = samples obtained after immunization.(2,6 = pORF-hFlk-1 LDC; 3,7 = pORF-mFlk-1 LDC; 4,8 = pORF-mcs LDC; 5,9 = GS)

To explore the possible mechanism by which the anticancer activity was induced by human VEGFR-2 LDC, we identified auto-antibodies against Flk-1 in the immunized mice. Sera from human VEGFR-2 LDC-immunized mice recognized VEGFR-2 protein on Western blots and by ELISA following immunization (Figure 4). In contrast, the sera isolated from controls showed negative staining.

### Inhibition of angiogenesis

**Figure 5:**
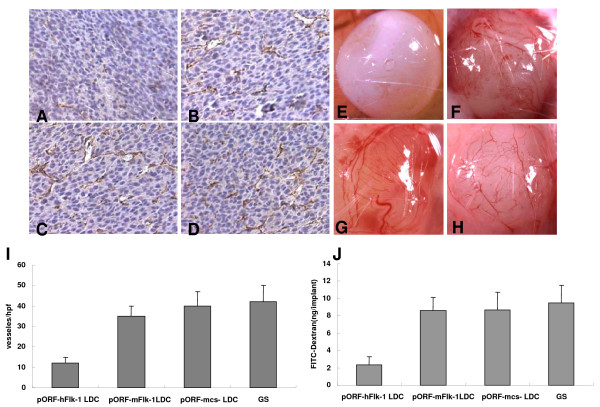
**Inhibition of angiogenesis within tumors**. BALB/c mice were immunized with pORF-hFlk-1 LDC, pORF-mFlk-1 LDC, pORF-mcs LDC or GS once a week for 6 weeks. The mice were then inoculated with 2 × 10^5 ^CT26 tumor cells. Frozen sections of tumor tissue were tested by immunohistochemical analysis with anti-CD31 antibody (A-D). Vessel density in tumor tissues from human VEGFR-2 LDC immunized mice indicated a significant decrease compared with controls (I; P < 0.01). Columns: means; bars: SD. Vascularization of alginate implants. Mice were immunized as above and alginate beads containing 1 × 10^5 ^CT26 tumor cells per bead were then implanted s.c. into the backs of mice 7 days after the last immunization. Beads were surgically removed 12 days later (E-H), and FITC-dextran was quantified (J). Bead uptake in mice immunized with human VEGFR-2 LDC showed a significant decrease compared with controls (P < 0.01). Columns: means; bars: SD.

Vaccination with human VEGFR-2 LDC resulted in the apparent inhibition of angiogenesis in tumors (Figure 5A) compared with control groups (Figure 5B–D). The number of microvessels also showed a significant decrease in sections stained with an antibody reactive to CD31 (Figure 5I). In addition, inhibition of angiogenesis could also be detected in the alginate-encapsulate tumor cell assay. Angiogenesis in alginate implants was quantified by measuring the uptake of FITC-dextran into the beads. Vascularization of alginate beads was apparently reduced, and FITC-dextran uptake was significantly decreased in human VEGFR2 LDC-immunized mice when compared with controls (Figure 5E–J). These results suggested that tumor angiogenesis was inhibited in human VEGFR-2 LDC-immunized mice, which resulted in the suppression of tumor growth.

### Assay of CTL-mediated cytotoxicity

**Figure 6:**
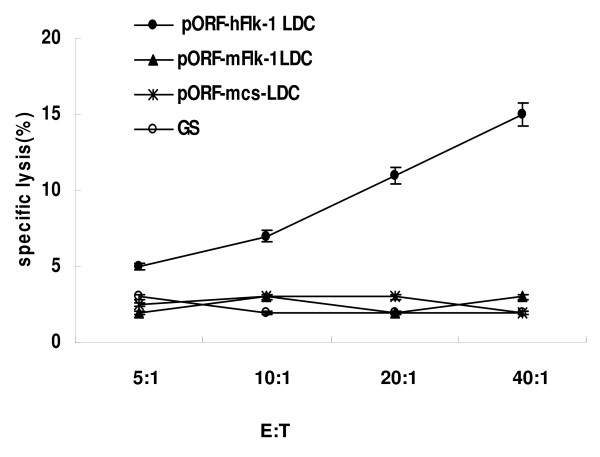
**CTL-mediated cytotoxicity in vitro**. T lymphocytes from mice immunized with pORF-hFlk-1 LDC, pORF-mFlk-1 LDC, pORF-mcs LDC or GS were tested against murine MS1 cells at different effector/target ratios by ^51^Cr release assay. T cells isolated from mice immunized with pORF-hFlk-1 LDC showed increased cytotoxicity against Flk-1-positive target MS1 cells (*P *< 0.05). Points: means of triplicate samples from one representative experiment; bars: SE.

Specific CTL activity was assayed by ^51^Cr release. These assays showed that T lymphocytes from the mice immunized with human VEGFR-2 LDC were more cytotoxic to hFlk-1+ MS1 cells than control groups (Figure 6). These findings indicate that both humoral and cellular immunity were mediated against hFlk-1.

### Observations of potential toxicity

**Figure 7:**
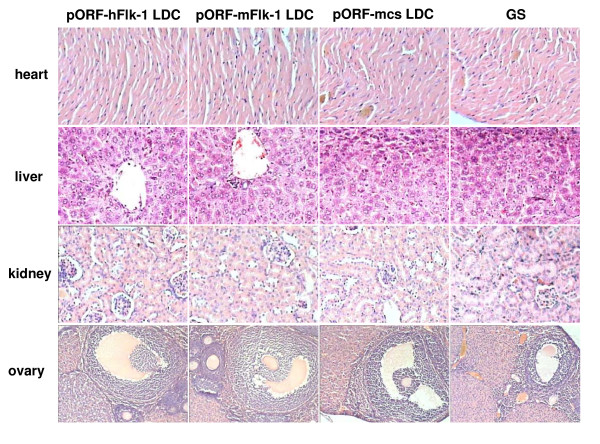
**Toxicity observation**. H & E staining of heart, liver, kidney and ovary in recipient mice. No hemorrhage in organs appeared in the pORF-hFlk-1 LDC group and no differences were seen among groups (pORF-hFlk-1 LDC, pORF-mFlk-1 LDC, pORF-mcs LDC, GS).

Vaccinated mice without tumors were investigated for more than 5 months for potential toxicity caused by the injected DNA. Spleen enlargement was observed in most mice, but no pathological changes to the liver, kidney, heart or ovary were found. No adverse consequences were indicated in gross measures such as weight loss, ruffling of fur, life span, behavior or feeding in the mice. No other pathological changes to the liver, kidney, heart or ovary were found by microscopic examination of tumor-bearing mice over a period of more than 5 months, but weight loss, ruffling of fur, behavior or feeding were changed (Figure 7). Only mild changes in peripheral blood counts and differentials were observed following the immunizations, while biochemical tests were normal (data not shown).

## Discussion

Immunization with LDC is an effective strategy for generating therapeutic anti-cancer immunity mediated by plasmid DNA. The enhanced anti-cancer activity elicited by LDC immunization is mediated in part by the generation of CD8^+ ^T cells with increased functional activity[15]. The breaking of immune tolerance to "self-antigens" associated with angiogenesis by active immunity[19] is an attractive approach to cancer therapy. Angiogenesis is a complex process involving many molecules and cellular events. VEGF and its receptor, VEGFR-2 (Flk-1/KDR), play a key role in tumor angiogenesis. That blocking the VEGF-VEGFR-2 pathway may inhibit tumor growth is exemplified in studies using neutralizing KDR/Flk-1 mAb[25], KDR/Flk-1 kinase inhibitors[26,27], or a dominant-negative Flk-1 receptor[28], all of which were shown to inhibit angiogenesis and tumor growth. It has been reported that peripheral T-cell tolerance against murine Flk-1 can be broken by an oral DNA vaccine encoding autologous Flk-1, delivered by an attenuated strain of *Salmonella typhimurium*[29]. Plasmid DNA vaccines are attractive because they are relatively easy to engineer and produce, and are safe to administer to humans [8-10].

Our studies demonstrate that immune tolerance can be broken by LDC-mediated immunization with human VEGFR-2. Using this approach, we induced anti-tumor effects in mouse models of colon carcinoma (CT26 cells) and breast cancer (4T1 cells), and against both primary tumors and lung metastases (4T1 tumors). A 50% molar change on the liposomes significantly increased the accumulation of DNA in the lungs of mice 24 h post-injection[30]. Thus, for lung metastases, this could be of great benefit. There was an apparent protection from tumor growth in mice immunized with human VEGFR-2 LDC.

The possible mechanism by which anti-cancer activity is induced by xenogeneic molecules has been reported previously[18,19]. Autoantibodies against mFlk-1 produced by mice immunized with human VEGFR-2 LDC were identified by Western blot analysis. T cells isolated from mice immunized with human VEGFR-2 LDC showed increased cytotoxicity against hFlk-1-positive MS1 target cells. In our studies, we also observed potential toxicity. Vaccination prolonged the lifespan of mice and improved the quality of life (QOL) of tumor-bearing mice. Weight loss, ruffling of fur, behavior, or feeding were changed in tumor-bearing mice post-treatment but improved between treatments. No pathological changes in the liver, kidney, ovary or heart were observed. Only mild changes of peripheral blood counts and differentials were observed following immunizations, and biochemical tests were normal. Thus, the human VEGFR-2 vaccine was well tolerated by mice.

## Conclusion

The enhanced anticancer activity elicited by human VEGFR-2 LDC immunization was dependent on the impairment of the formation of new tumor blood vessels through an anti-angiogenesis mechanism. In addition, immune tolerance to self-Flk-1 was consequently broken down by vaccination with human VEGFR-2 LDC. The human VEGFR-2 vaccine was well tolerated by mice. Our data may provide a vaccine strategy for cancer therapy, through the induction of autoimmunity against the growth factor receptor associated with angiogenesis mediated by plasmid DNA encoding a xenogeneic homologue.

## Abbreviations

VEGF: vascular endothelial growth factor; VEGFR-2: vascular endothelial growth factor receptor-2; LDC: liposome-DNA complexes; DC: dendritic cell; SD: Sprague-Dawley; QOL: quality of life.

## Competing interests

The authors declare that they have no competing interests.

## Authors' contributions

KX, RZB and YW performed the experiments; KX drafted the manuscript; RZB and YW contributed to the manuscript; QL and KL performed the statistical analysis and helped to draft the manuscript. YQW conceived the study, and participated in its design. All authors have read and approved the final manuscript.
